# Case Report: Delayed non-calculous biliary obstruction after left hepatectomy associated with intraductal necrotic material with a biliary epithelial component

**DOI:** 10.3389/fmed.2026.1967233

**Published:** 2026-10-12

**Authors:** Ye Wang, Shengchen Nan, Xu Jiang, Xinglin Jin

**Affiliations:** Department of Hepatobiliary Surgery, Yanbian University Hospital, Yanji, Jilin, China

**Keywords:** biliary epithelial component, delayed biliary obstruction, ERCP, hepatectomy, intraductal necrotic material, obstructive jaundice

## Abstract

Delayed biliary obstruction after hepatectomy is usually attributed to calculi, strictures, or recurrent malignancy. Intraductal necrotic material of biliary epithelial origin is uncommon and may mimic sludge or stones on imaging. A 70-year-old woman with a 15-year history of hepatitis C underwent left hepatectomy and cholecystectomy for a cirrhotic nodule at another institution. Five months later, she developed progressive jaundice, intermittent abdominal pain, and fever. MRCP and contrast-enhanced CT showed biliary dilatation with sludge-like or heterogeneous material in the common bile duct. ERCP revealed dark-red, soft intraductal material rather than calculi. After balloon dilation, forceps sampling at three intraductal sites, and multiple basket and balloon sweeps, the material was removed. Histology showed extensive necrosis and degenerated epithelioid cells, and CK7 and CK19 positivity supported a biliary epithelial component. Completion cholangiography showed free drainage without an obvious residual stricture. By post-ERCP day 3, total bilirubin had decreased to 114 μmol/L. At 1-month follow-up, jaundice had markedly subsided, serum transaminases, bilirubin, and alkaline phosphatase were within reference ranges, and no adverse symptoms were reported. This case highlights a rare non-calculous cause of delayed biliary obstruction after hepatectomy. When postoperative imaging suggests sludge or stones, ERCP can clarify the nature of the intraductal material, obtain tissue for pathological characterization, and restore biliary drainage.

## 1 Introduction

Jaundice with biliary dilatation after hepatectomy requires prompt evaluation for calculi, a benign stricture, postoperative biliary injury, and tumor-related obstruction (1, 2). Cross-sectional imaging can demonstrate ductal dilatation and intraductal abnormalities, but sludge, casts, clot, necrotic tissue, and stones may overlap in appearance.

Biliary epithelial injury and impaired microvascular perfusion can lead to epithelial necrosis, sloughing, and intraductal obstruction (3, 4). Biliary cast syndrome is best recognized after liver transplantation, although similar obstructing material has been described in nontransplant settings (5–12). The relationship between delayed non-calculous obstruction and previous hepatectomy remains poorly characterized.

We report a patient who developed delayed non-calculous biliary obstruction after left hepatectomy. Imaging suggested sludge-like material, whereas ERCP unexpectedly retrieved extensive dark-red necrotic material. Histology and CK7/CK19 immunoreactivity supported a biliary epithelial component, and endoscopic clearance was followed by clinical and biochemical improvement.

## 2 Case presentation

A 70-year-old woman with a 15-year history of hepatitis C underwent left hepatectomy and cholecystectomy at another institution 5 months before admission for a cirrhotic nodule. The operative report for the previous left hepatectomy was unavailable. The outside pathology report documented a left hemihepatectomy specimen measuring 15.5 × 10 × 5 cm and diagnosed chronic hepatitis with nodular cirrhosis. Marked lymphocytic infiltration was present in the fibrous septa, and some bile ducts were dilated and showed hemorrhage. No definite neoplastic change was identified. Immunohistochemistry showed negative CD10 expression, CD34 positivity in vascular structures, HepPar-1 positivity, and a Ki-67 labeling index of approximately 1%. No postoperative complications were reported in the available medical records.

The patient developed progressive yellowing of the skin, mucosa, and sclera over 2 weeks, accompanied by intermittent abdominal pain. Fever began 3 days before admission and reached 40.0 °C. No further high fever was recorded after admission.

On admission, total bilirubin was 234.0 μmol/L (reference range, 3–22), direct bilirubin was 182.4 μmol/L (0–7), alkaline phosphatase was 155 U/L (42–140), gamma-glutamyl transferase was 172 U/L (8–58), AST was 88 U/L (14–36), and ALT was 64 U/L (<35). C-reactive protein was 188.60 mg/L (0.5–10), and procalcitonin was 9.49 ng/mL (0.1–0.25). The leukocyte count was 4.10 × 10^9/L (4–10), with 93.8% neutrophils (50–70). Albumin was 38 g/L (35–50), INR was 1.11 (0.76–1.15), prothrombin time was 12.9 s (9–15), platelets were 76 × 10^9/L (100–300), CA 19-9 was 5.3 U/mL (0–34), creatinine was 73 μmol/L (46–92), and lactate was 0.8 mmol/L (0.5–1.6). Blood and bile cultures were not performed.

MRCP showed marked intrahepatic and extrahepatic biliary dilatation with sludge-like material in the common bile duct (Figures 1A,B). On review of the available images, the maximal common bile duct diameter was approximately 19 mm, and the most dilated intrahepatic bile duct measured approximately 9 mm. Contrast-enhanced CT further demonstrated heterogeneous intraductal density within the common bile duct. The hepatic artery supplying the remnant liver was patent, without visible stenosis, occlusion, or thrombosis.

**Figure 1:**
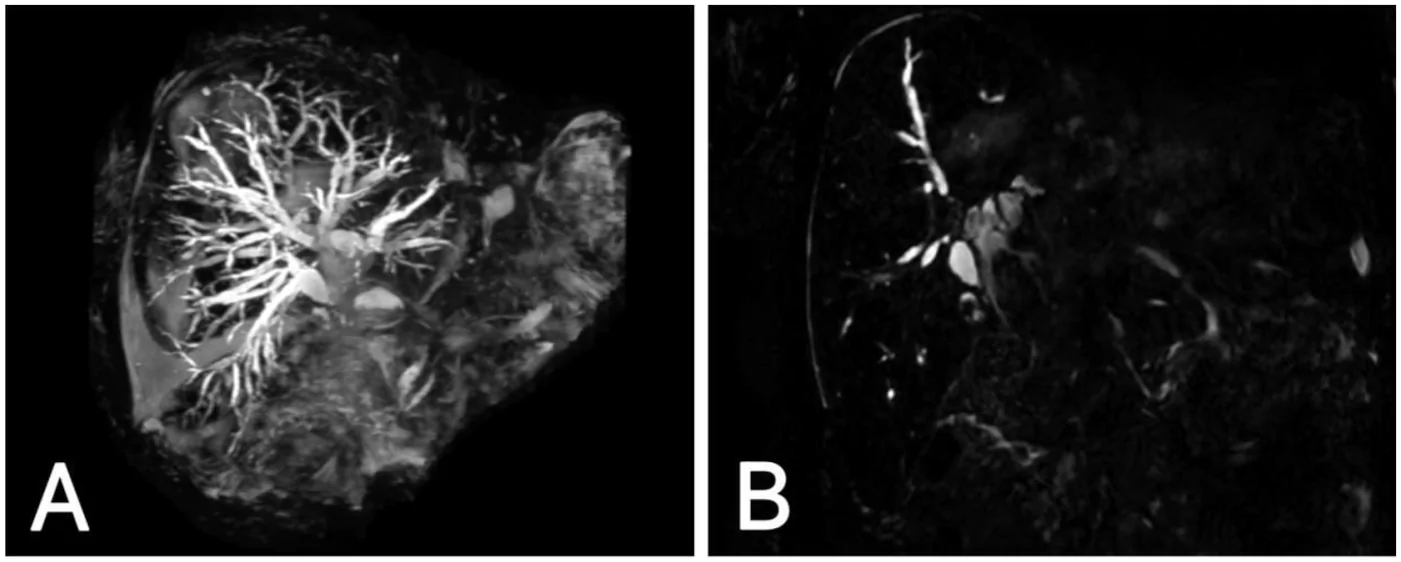
Magnetic resonance cholangiopancreatography. Coronal reconstructions **(A,B)** show marked intrahepatic and extrahepatic biliary dilatation and sludge-like material in the common bile duct. The maximal common bile duct diameter was approximately 19 mm, and the most dilated intrahepatic bile duct measured approximately 9 mm.

Fever with elevated inflammatory markers, jaundice, hyperbilirubinemia, and imaging evidence of biliary obstruction met the Tokyo Guidelines 2018 criteria for definite acute cholangitis and supported at least Grade II severity (1). Cefoperazone sodium–sulbactam sodium was administered during the perioperative period for a total of 7 days. These findings provided the clinical context for urgent biliary decompression (1, 2); the principal diagnostic question was the nature of the obstructing material.

ERCP was performed for suspected obstruction by sludge-like material and for biliary decompression. Endoscopic sphincterotomy was not performed. After cholangiography, the common bile duct was dilated using a 10-mm balloon dilation catheter. Initial cholangiography showed a common bile duct filling defect and impaired drainage (Figure 2A). Forceps samples were obtained from three different intraductal sites, with an aggregate sample size of approximately 0.5 × 0.5 × 0.3 cm. Multiple sweeps with a retrieval basket and balloon removed a large amount of dark-red, soft material (Figure 3). No typical calculi were identified. After removal of the material, purulent bile drained through the papilla. Completion cholangiography showed free flow of contrast into the duodenum, without an obvious residual stricture or filling defect (Figure 2B). A nasobiliary drain was placed temporarily.

**Figure 2:**
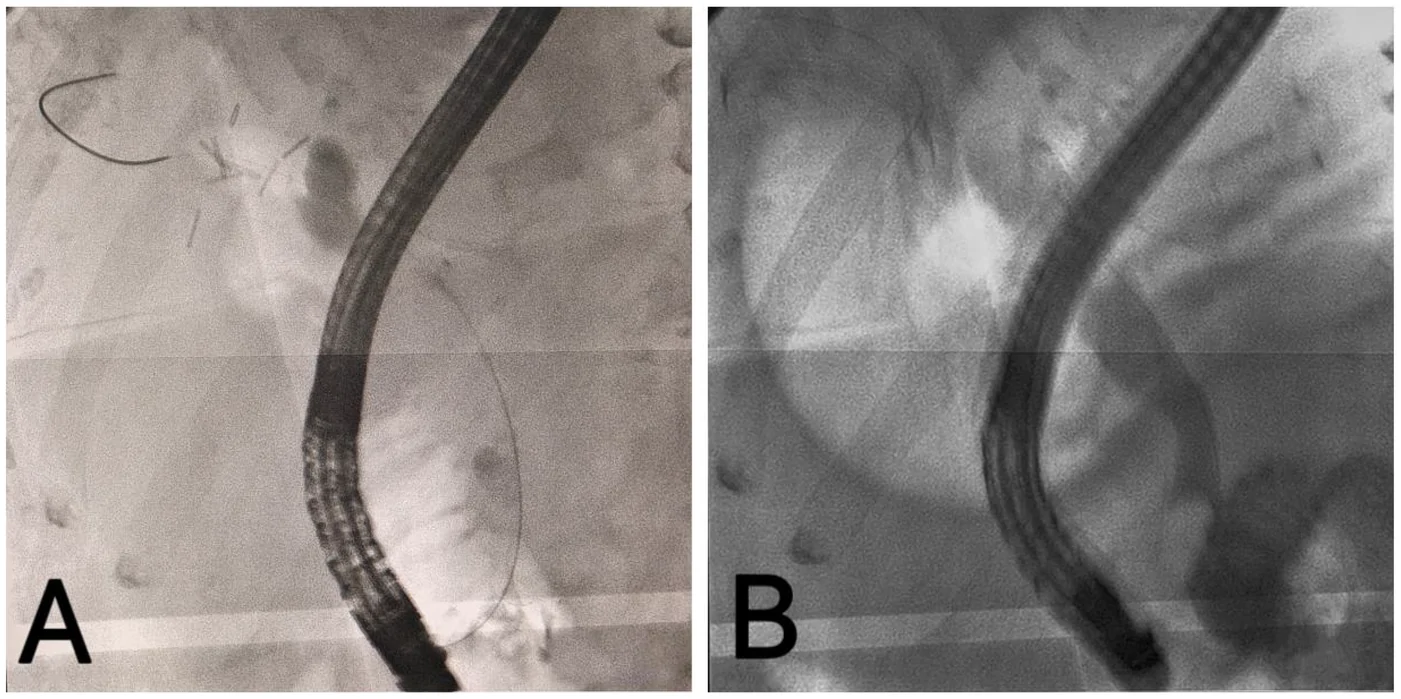
Endoscopic retrograde cholangiopancreatography. Initial cholangiography **(A)** shows a common bile duct filling defect and impaired drainage. After extraction **(B)**, no residual filling defect or obvious stricture remained, and contrast drained freely into the duodenum.

**Figure 3:**
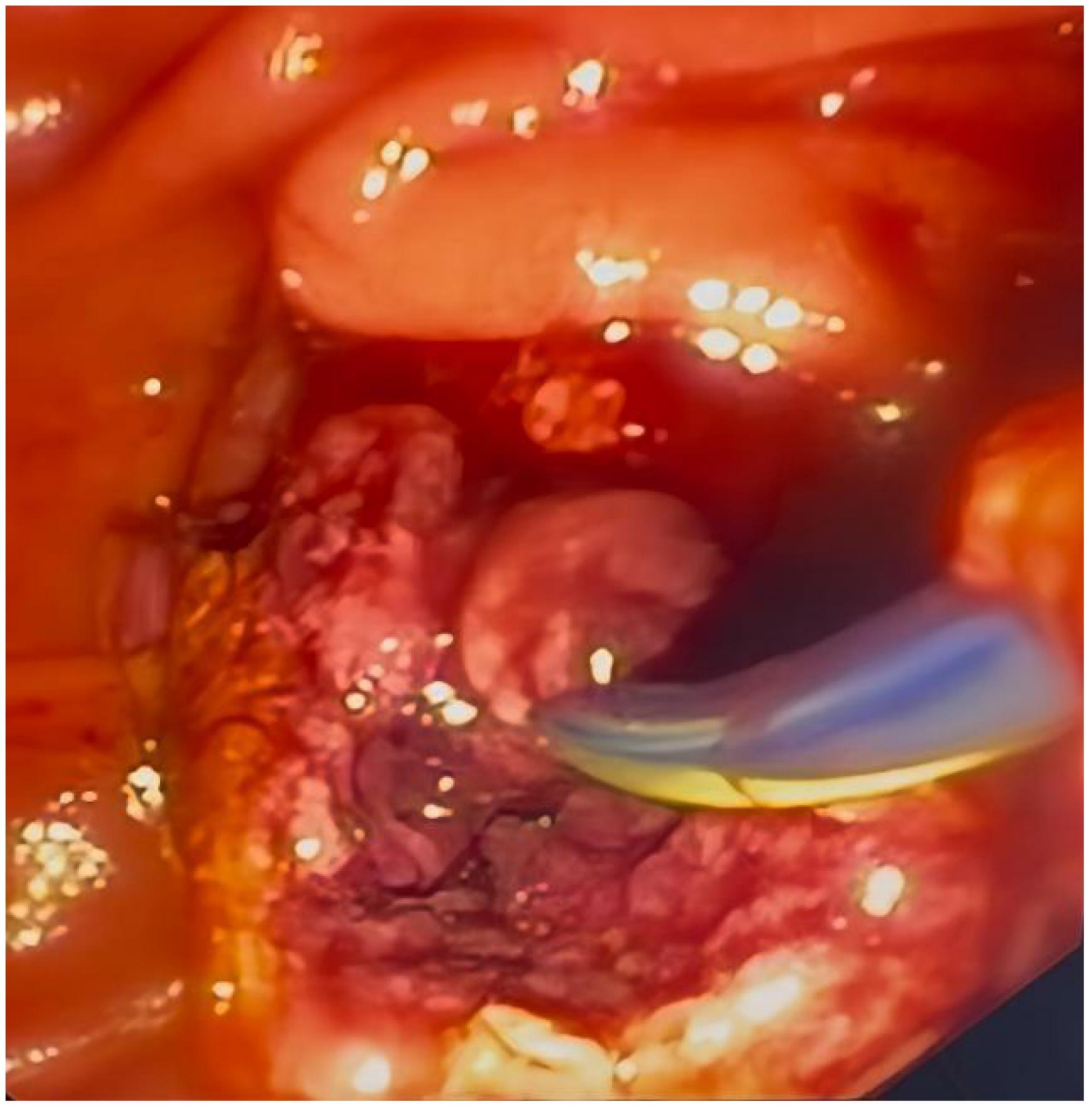
Endoscopic appearance of the retrieved material. A large amount of dark-red, soft intraductal material was removed with a retrieval basket and balloon; no typical calculi were identified.

Histopathological examination of the retrieved material showed extensive necrosis and degenerated epithelioid cells (Figure 4). Immunohistochemical staining was positive for CK7 and CK19, supporting a biliary epithelial component in the retrieved necrotic material.

**Figure 4:**
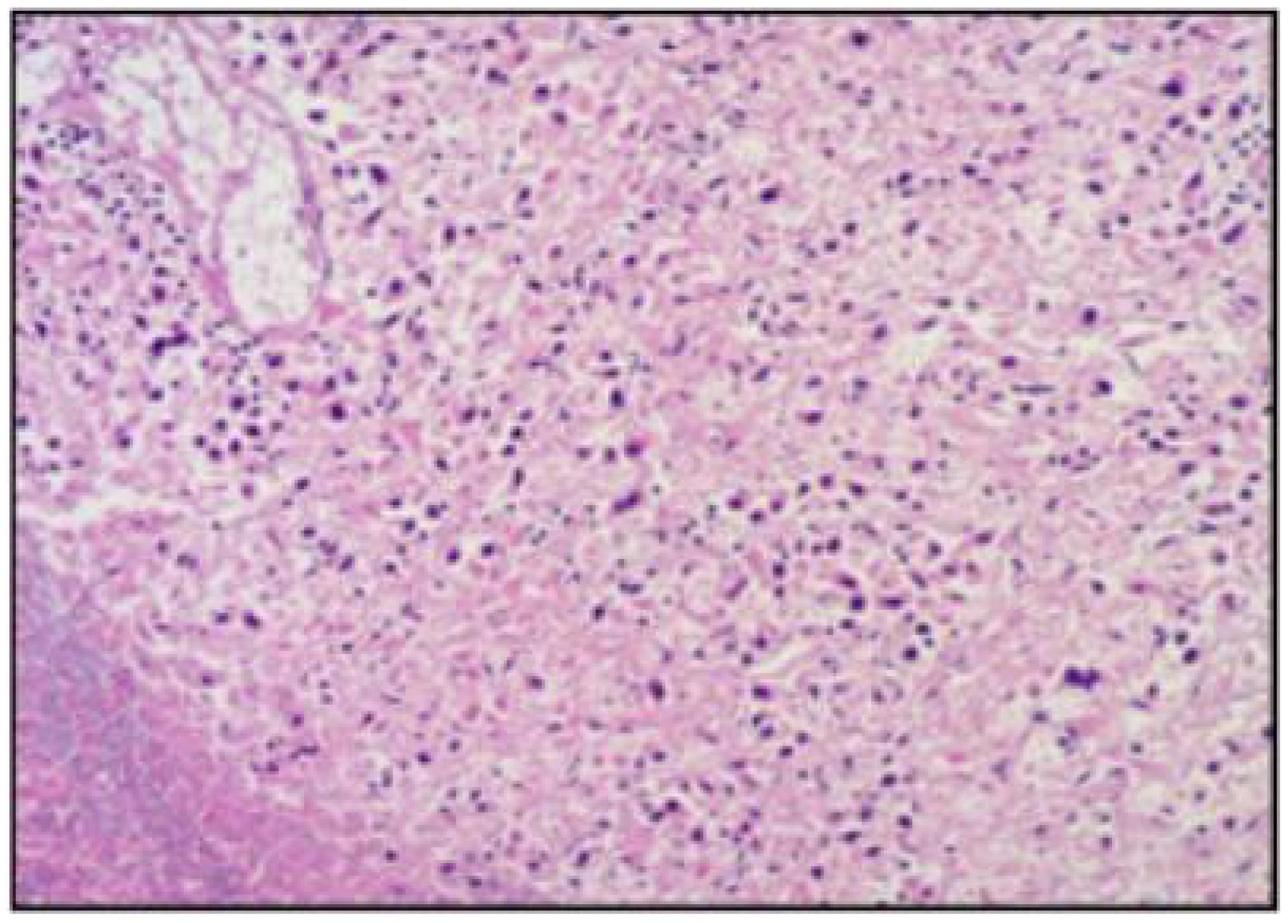
Histopathology of the retrieved intraductal material. Hematoxylin and eosin staining (×100) shows extensive necrosis and degenerated epithelioid cells.

On post-ERCP day 1, the nasobiliary drain remained patent and produced clear golden bile. The patient had no fever or abdominal pain, and serum amylase was within the reference range. Total bilirubin was 207.2 μmol/L, direct bilirubin was 182.1 μmol/L, and alkaline phosphatase transiently increased from 155 to 319 U/L. The drain was removed. By post-ERCP day 3, jaundice had visibly improved; total bilirubin was 114 μmol/L, direct bilirubin was 100 μmol/L, AST was 52 U/L, and ALT was 40 U/L. At 1-month follow-up, jaundice had markedly subsided, serum transaminases, bilirubin, and alkaline phosphatase were within reference ranges, and no adverse symptoms were reported. The clinical course is summarized in Figure 5.

**Figure 5:**
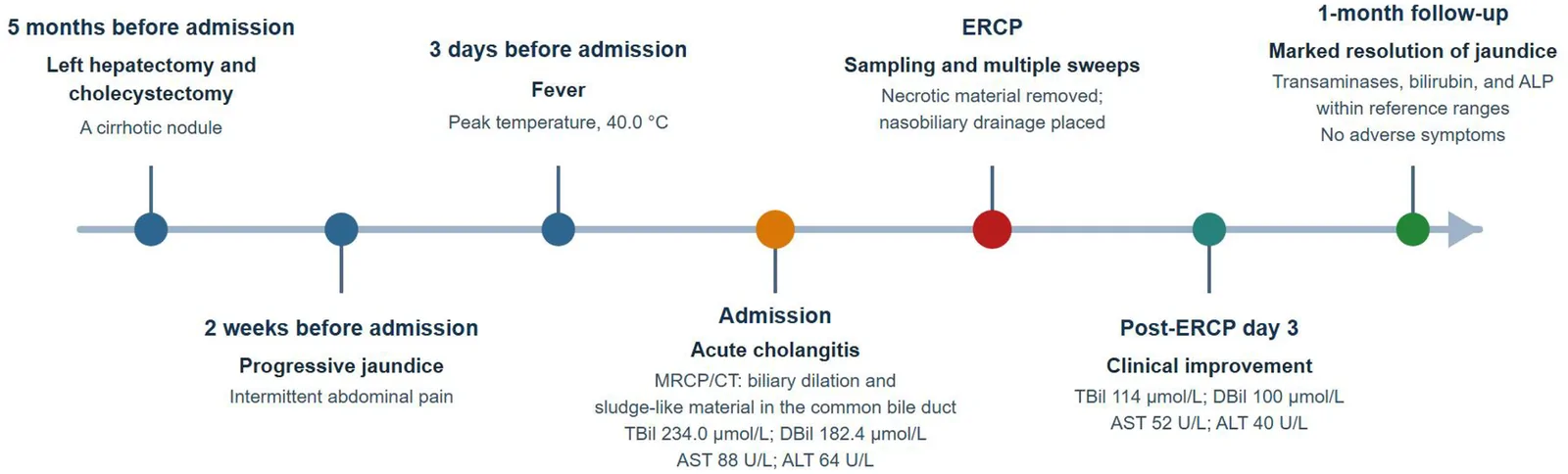
Clinical timeline.

## 3 Discussion

The principal finding in this case was the nature of the obstructing material. Although imaging suggested sludge-like material or stones, ERCP demonstrated extensive dark-red, soft, non-calculous material. Histology showed extensive necrosis, and CK7 and CK19 positivity supported a biliary epithelial component. ERCP therefore refined the initial imaging impression and provided both tissue sampling and biliary decompression.

Biliary cast syndrome was considered in the differential diagnosis. Classic casts are tubular or branching structures that conform to the biliary tree and may contain necrotic epithelium, bile pigment, and inflammatory material (5–10). Nontransplant casts have also been described after cholangitis and other biliary insults (8, 11, 12). In this patient, the retrieved material was dark red, soft, and not a typical formed cast. The differential also included organized clot, inflammatory debris, sloughed tissue, and necrotic neoplastic material. The absence of typical calculi, the outside pathology showing no definite neoplastic change, and CK7/CK19 positivity favored the interpretation of necrotic material with a biliary epithelial component. This presentation may lie within the broader spectrum of epithelial injury and cast-like obstruction without meeting the conventional morphological description of a biliary cast.

The 5-month interval supports a temporal association between the previous left hepatectomy and the delayed biliary obstruction, but does not establish causation. Possible contributors include postoperative biliary epithelial injury, local microvascular compromise, impaired epithelial repair, bile stasis, and postoperative benign narrowing (3, 4, 13). The operative report for the previous left hepatectomy was unavailable, so the event cannot be attributed to a specific vascular, thermal, or biliary injury. The patent hepatic artery on pre-ERCP imaging argued against ongoing large-vessel arterial compromise, although a transient or localized ischemic insult associated with the previous hepatectomy could not be assessed retrospectively. Chronic hepatitis C and cirrhosis may have increased susceptibility through impaired microcirculation and tissue repair, but they are considered possible predisposing conditions rather than proven causes.

ERCP served both diagnostic and therapeutic roles in this case. Multiple basket and balloon sweeps removed the intraductal material, and completion cholangiography showed free drainage without an obvious residual stricture. Fever and abdominal pain resolved, while bilirubin and aminotransferase levels decreased substantially by post-ERCP day 3, supporting the retrieved material as a major contributor to the mechanical obstruction. Although alkaline phosphatase transiently increased on post-ERCP day 1, the measured liver tests were within reference ranges at 1 month, accompanied by marked resolution of jaundice.

The unavailable operative report from the previous left hemihepatectomy, the absence of microbiological cultures, and the lack of longer-term imaging follow-up limited assessment of the underlying mechanism and long-term outcome. These limitations did not affect the direct endoscopic, histological, and immunohistochemical characterization of the obstructing material.

## 4 Conclusion

Intraductal necrotic material with a biliary epithelial component is an unusual cause of delayed non-calculous biliary obstruction after hepatectomy. When postoperative imaging suggests sludge or stones, ERCP with tissue sampling can clarify the nature of the obstruction and restore biliary drainage. The temporal relationship supports a possible association with the prior hepatectomy. Follow-up is required to assess long-term biliary patency and the recurrence of obstruction or biliary stricture.

## 5 Patient perspective

At 1-month follow-up, the patient reported marked improvement in jaundice and no adverse symptoms. She remained willing to continue follow-up.

## Data Availability

The original contributions presented in the study are included in the article/Supplementary Material, further inquiries can be directed to the corresponding authors.
